# TiO_2_@PEI-Grafted-MWCNTs Hybrids Nanocomposites Catalysts for CO_2_ Photoreduction

**DOI:** 10.3390/ma11020307

**Published:** 2018-02-20

**Authors:** Caterina Fusco, Michele Casiello, Lucia Catucci, Roberto Comparelli, Pietro Cotugno, Aurelia Falcicchio, Francesco Fracassi, Valerio Margiotta, Anna Moliterni, Francesca Petronella, Lucia D’Accolti, Angelo Nacci

**Affiliations:** 1 ICCOM-CNR (Istituto di Chimica dei Composti OrganoMetallici-Consiglio Nazionale delle Ricerche), SS Bari, Via Orabona 4, 70126 Bari, Italy; 2 Dipartimento di Chimica, Università di Bari “A. Moro”, Via Orabona 4, 70126 Bari, Italy; michele.casiello@uniba.it (M.C.); lucia.catucci@uniba.it (L.C.); pietro.cotugno@uniba.it (P.C.); francesco.fracassi@uniba.it (F.F.); 3 IPCF-CNR (Istituto per i Processi Chimico Fisici–Consiglio Nazionale delle Ricerche), SS Bari, Via Orabona 4, 70126 Bari, Italy; r.comparelli@ba.ipcf.cnr.it (R.C.); v.margiotta@ba.ipcf.cnr.it (V.M.); f.petronella@ba.ipcf.cnr.it (F.P.); 4 CNR-IC (Consiglio Nazionale delle Ricerche–Istituto di Cristallografia), Via Amendola 122/O, 70126 Bari, Italy; a.falcicchio@ic.cnr.it (A.F.); a.moliterni@ic.cnr.it (A.M.)

**Keywords:** artificial photosynthesis, capture and valorization of CO_2_, MWCNTs hybrids nanocomposites

## Abstract

Anatase (TiO_2_) and multiwalled carbon nanotubes bearing polyethylenimine (PEI) anchored on their surface were hybridized in different proportions according to a sol-gel method. The resulting nanocomposites (TiO_2_@PEI-MWCNTs), characterized by BET, XRD, XPS, SEM, and UV techniques, were found efficient catalysts for CO_2_ photoreduction into formic and acetic acids in water suspension and under visible light irradiation. PEI-grafted nanotubes co-catalysts are believed to act as CO_2_ activators by forming a carbamate intermediate allowing to accomplish the first example in the literature of polyamines/nanotubes/TiO_2_ mediated CO_2_ photoreduction to carboxylic acids.

## 1. Introduction

During the past decade, great deal of attention has been dedicated to converting solar energy into chemical energy in the form of so-called “solar fuels”, such as H_2_, methanol, methane, formic acid, etc. In this context, the reduction of carbon dioxide is gaining more and more importance in the research area of chemistry and materials, not only for solving the problems resulting from environmental pollution, but also for finding ways to maintain the carbon resources which are being depleted by the burning of fossil fuels [1].

A very promising method is the photoreduction of CO_2_ to produce clean hydrocarbon fuels under sunlight irradiation, a process that mimics the natural photosynthesis in plants. Among all photocatalysts, anatase TiO_2_ still remains the most promising candidate considering its photostability, corrosion resistance, low cost, and strong oxidation power [2,3,4].

However, photocatalytic efficiency of TiO_2_ is limited due to its large band gap (3.2 eV), which can only be excited under ultraviolet irradiation and rapid recombination of photoinduced electrons and holes. To solve these problems, a variety of dopants has been investigated, ranging from transition metals (e.g., Pd, Pt, Fe, etc.) [5,6,7,8,9,10] and their oxides (e.g., Cu_2_O, CeO_2_, etc.), [11] to non-metal ions (N and I) [12] and rare hearth derivatives (La_2_O_2_) [13].

During the past decade, there has been a growing interest in hybridizing TiO_2_ with carbon nanostructured materials, such as nanotubes (CNTs) [14,15,16,17,18] and graphene [19], that play the role of transferring photogenerated electrons from the TiO_2_, suppressing the recombination rate of the electron–hole pairs. Recently, a new strategy is emerging concerning the modification of TiO_2_ with co-catalysts capable of enhancing CO_2_ adsorption [20]. Main approaches are based on metal oxides (mainly MgO) [21,22], metal organic framework (MOFs) [23] and amines [24,25].

In this context, being carbon nanotubes amongst the most promising solid sorbents for CO_2_ capture [26,27], in particular when their walls are decorated with CO_2_-philic groups [28,29,30,31,32], we sought to enhance photocatalytic performance of titania by means of its conjugation with carbon nanotubes bearing polyamines anchored on their surface, with the specific task of adsorbing the CO_2_ reactant and help the redox process.

With this in mind, in our ongoing efforts aimed at searching new green catalytic methods [33,34,35,36,37,38], we prepared a series of hybridized materials based on anatase and amine-grafted multiwalled carbon nanotubes (TiO_2_@amine-MWCNTs) and tested their ability to serve as an efficient catalyst for photoreduction of carbon dioxide.

## 2. Materials and Methods

### 2.1. Chemicals

Common commercial solvents and reagents of high purity were employed. Commercial Caroat^®^ (Pullach, Germany) triple salt (2KHSO_5_·KHSO_4_·K_2_SO_4_), a gift from Peroxid-Chemie (Degussa, Germany), was the source of potassium monoperoxysulfate (caroate) for the synthesis of TFDO [39,40]. MWCNTs (purity >90 wt.%, diameter between 0.7 and 1.4 nm) are commercially available from Sigma-Aldrich (Milan, Italy) in form of powder.

Carbon dioxide, O_2_, Argon and Helium were with purity of 99.99%, a gas burette (Figure S1) was used to measure gas volume in adsorption/desorption experiments. Formic and acetic acid analyses were performed with an Ion Chromatograph Dionex DX120 (Ion Pac ICE-AS6 Analytical Column, Monza, Italy); 0.8 mM heptafluorobutyric acid (Sigma-Aldrich) was used as eluent (flow 1.00 mL/min) and 5.0 mM tetrabutylammonium hydrate (Sigma-Aldrich) as regenerating eluent (flow 4.5 mL/min).

Formic acid 99% and acetic acid (Sigma Aldrich) were employed as standard for building IC calibration curves. Analyses were carried out by using a sample loop of 25 μL, with a column pressure less than 640 PSI and a background of 35–40 μS.

GC analyses for liquid and gas phase were performed with a GC Tracera Shimadzu 2010 (Milan, Italy) with a Detector BID-2010 (Shimadzu, Milan, Italy) plus and a Micropacked ST column (temperature program isotherm at 40 °C 30 min). Infrared analyses were performed in the range 400–4000 cm^−1^ (64 scans), using a PerkinElmer 2000 FT-IR spectrometer (Milan, Italy).

### 2.2. Physico-Chemical and Morphological Characterization of Nanocomposites

Thermogravimetric analyses were recorded on a TGA Q500 (TA Instruments, New Castle, GA, USA) using a flowing nitrogen atmosphere. The samples (about 1 mg per sample) were equilibrated at 100 °C for 20 min and then heated at a rate of 10 °C min^−1^ up to 800 °C.

Elemental analyses were performed on a Thermo Flash EA 1112 series CHNS-O elemental analyzer (Monza, Italy) with an accepted tolerance of 0.4 units.

TEM (Transmission electron microscopy) analyses were performed with a TEM Philips EM208 instrument (Vienna, Austria) using an accelerating voltage of 100 kV. The samples were prepared by dropping aliquots of the DMF dispersions onto TEM grids (200 mesh, Nichel, carbon only, Ted Pella, Milan, Italy).

BET (Brunauer-Emmett-Teller) specific surface area was obtained by N_2_ adsorption-desorption method on powder samples using a Micromeritics TriStar II 3020 (Micromeritics, Norcross, U.S.A.). The samples (500 mg) were pre-treated at 80 °C for 2 h before N_2_ adsorption.

FE-SEM investigations were performed by a Zeiss Sigma (Milan, Italy) microscope operating in the range 0.5–20 kV and equipped with an in-lens secondary electron detector. FE-SEM samples were prepared by sticking the powder on double sided carbon tape. Samples were mounted onto stainless-steel sample holders by using and grounded by silver paste.

UV-Vis diffuse reflectance spectra were recorded with a UV-vis-NIR Cary 5 (Varian, Palo Alto, CA, USA) spectrophotometer equipped with an integrating sphere (BaSO_4_).

X-Ray powder diffraction data were collected at room temperature by using an automated Rigaku RINT2500 laboratory diffractometer (50 KV, 200 mA, Neu-Isenburg, Germany) equipped with the silicon strip Rigaku D/teX Ultra detector. Detailed XRD analyses of hybrids nanocomposites are showed in Figure S2.

X-ray photoelectron spectroscopy (XPS) analyses were performed on a PHI VersaProbe II spectrometer (Phisical Electronics, Kanagawa, Japan) equipped with a monochromatic Al Kα X-ray source (1486.6 eV) operating at a spot size of 100 μm, corresponding to a power of 24.4 W. Survey (0–1400 eV) and high resolution (C 1*s*, O 1*s*, N 1*s*, Ti 2*p*, Cl 2*p*, F 1*s*) spectra were recorded in FAT (fixed analyzer transmission) mode at pass energy of 117.4 eV and 46.95 eV, respectively. All spectra were acquired at a take-off angle of 45° with respect to the sample normal. Dual-beam charge neutralization was constantly applied during analysis. Charge correction of the spectra was performed by taking the *sp*2 graphitic component of the C 1*s* spectrum as internal reference (binding energy, BE = 284.6 eV).

XPS analysis was repeated on three different spots for each sample. During analyses, special care was devoted to verifying that no change in the samples was induced by exposure to the X-ray beam and the charge neutralization dual-beam. Detailed spectra processing was performed by commercial MultiPak software (Version 9.5.0.8, 30-10-2013, Ulvac-PHI, Inc., Kanagawa, Japan). The high-resolution spectra were fitted with mixed Gaussian-Lorentzian peaks after a Shirley background subtraction.

The DO 9721 quantum photo-radiometer (Delta Ohm, Pordenone, Italy) and thermometer data logger were used for measuring illuminance, irradiance, luminance and temperature.

### 2.3. Preparation of ox-MWCNTs (**2**) with TFDO

Preparation was accomplished according to our previous method [41,42]. MWCNTs (600–1000 mg) were placed in CH_2_Cl_2_ (50–100 mL) and the mixture was sonicated for 30 min. An aliquot (35 mmol) of the standardized cold solution of methyl(trifluoromethyl)dioxirane (TFDO, 70 mL, 0.5 M) in 1,1,1-trifluoropropanone (TFP), was then added in one portion to the mixture, which was stirred and kept at 0 °C. After 24 h the reaction was complete, the mixture was filtered, and ox-CNTs **2** were washed several times with CH_2_Cl_2_ followed by MeOH, with recovery of 700–1300 mg.

### 2.4. Epoxide Ring Opening of ox-MWCNTs (**2**): Preparation of Amine-Grafted-MWCNTs **3** and **4**

This procedure was used for preparation of amine grafted-MWCNTs **3** and **4** [41,42]. Oxidized nanotubes 2 (1 g) were dispersed in 1,6-hexamethylenediamine (3.7 g) or branched polyethylenimine (5 g PEI MW 600, 99% of purity) and the mixture was heated at reflux at 140 °C under N_2_ for 12 h. Next, the reaction mixture was diluted with methanol (20 mL) and filtered through a PTFE membrane filter paper (0.2 μm). The solid was washed several times with CH_2_Cl_2_ followed by MeOH to remove any excess of amine leading to HMDA-MWCNTs (**3**, 1.20 g) or PEI -MWCNTs (**4**, 1.25 g). Elemental analyses for **3**: C (95.45%), H (3.24%), O (0.30%), N (1.01%); for **4**: C (91.59%), H (5.61%), O (0.17%), N (2.63%).

### 2.5. Preparation of hybrids nanocomposites photocatalysts **1A**, **1C**, **4A**-**C**

Preparation of hybrids nanocomposites was accomplished to previous method [14], titanium tetrachloride (98% TiCl_4_), hydrochloric acid (38% HCl) and distilled water with a molar ratio of TiCl_4_:HCl:H_2_O = 0.1:1:200 were homogenized under magnetic stirring for 30 min. The thus obtained transparent mother solution was then used as the raw material for preparing nanocomposites for photocatalytic experiments. Proper amounts of both p-MWCNTs (1) and functionalized PEI-MWCNTs (4) were separately dispersed into the proper volume of mother solution according to Table 1.

After suspension, mixture was sonicated and then ammonia was slowly added (until neutrality) under vigorous stirring, thus inducing TiO_2_ precipitation and formation of random agglomerates by inclusion of CNTs [14]. After filtration, nanocomposites **1A**, **1C** and **4A**-**C** were washed with distilled water and dried at 80 °C for 24 h.

### 2.6. Photocatalytic Experiments

The photocatalytic activities of samples **1A-C**, **4A**-**C** were evaluated by the reduction of CO_2_ in H_2_O [9,43] (see Section 3.3). The apparatus used for the photocatalytic experiments consists of a three necked Pyrex batch photoreactor of cylindrical shape containing 20 mL of aqueous suspension. The photoreactor was provided with a jacket for cooling water circulation and ports in its upper section for the inlet and outlet of gases, for sampling and for pH and temperature measurements. A HRC UV-VIS lamp 300 W (Sanolux, Milan, Italy) and Xe-Halogen lamp 400 W (Radium, Milan, Italy) were placed in proximity (5–6 cm) of the reactor. Argon was bubbled for 30 min, to avoid the presence of air, and then CO_2_ for approximately 60 min before switching on the lamps. The amounts of catalyst used were 150 and 500 mg in 20 mL of solution (loading of 7.5 and 25 mg/mL). The initial pH was 4.58. The temperature inside the reactor was held at approximately 25 °C by a continuous circulation of water in the jacket around the photoreactor. The photoreactivity runs lasted in 5.0 h and the products were detected by Ion Cromatography and GC-BID.

### 2.7. UBA Reduction Experiments

Experiments were performed following a protocol previously reported in literature [18,44,45]. The proper amount of the TiO_2_/MWCNTs powder was dispersed in a mixture of chloroform (61%) hexane (27%) and ethanol (12%), obtaining a concentration of 5000 ppm. Finally, the required volume of a stock solution of UBA in EtOH (2.5 × 10^−3^ M) was added to reach the desired CHCl_3_:Hexane: EtOH ratio, and fix the final UBA concentration at 10^−5^ M. Such a reaction mixture was poured in a quartz cuvette, sealed by a Teflon-faced rubber cap and purged under N_2_ flow for 20 min, under stirring. Such a procedure has a twofold role: (i) to remove oxygen from the reaction environment and (ii) to promote the absorption-desorption equilibria between the photocatalyst and the UBA. Next, the mixture, was irradiated under continuous stirring with UV light using a medium pressure 200 W mercury lamp (λ > 250 nm), equipped with neutral density filters in order to achieve a light flux of 5 mW/cm^2^. At scheduled time intervals, the cuvette was removed from the light source and the reaction mixture was transferred in a centrifuge tube under inert atmosphere. The UBA/photocatalyst suspension was separated by centrifugation, and the liquid phase analyzed by absorption spectroscopy. The reaction was monitored by recording difference absorption spectra of the irradiated reaction mixtures. Any change in the absorption line-shape was then clearly identified for each sample, by considering as reference the respective unphotolyzed solution.

## 3. Results and Discussion

### 3.1. Synthesis and Characterization of Amine-Grafted-MWCNTs

Grafting of amines onto the carbon nanotubes were performed with a recent approach based on epoxidation of CNTs followed by nucleophilic oxirane ring opening [41,42]. This strategy is a greener alternative to the most used method via chemical oxidation with strong acids (H_2_SO_4_/HNO_3_), that tends to damage the graphite-like structure of nanotubes [31,46].

**Figure 1:**
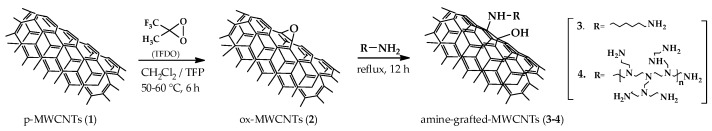
Synthesis of amine-grafted carbon nanotubes **3**-**4**.

The milder functionalization was accomplished by treatment of pristine nanotubes **1** (p-MWCNTs) with methyl (trifluoromethyl) dioxirane (TFDO), a well-known oxidant suitable for efficient and selective epoxidations of alkenes under mild and neutral conditions. Next, epoxidized nanotubes **2** (ox-MWCNTs) were subjected to the oxirane ring opening reaction with two different polyamines, namely 1,6-hexamethylendiamine (HMDA) and branched polyethylenimine (PEI), affording the corresponding amine-grafted MWCNTs **3**-**4** (Figure 1).

**Figure 2:**
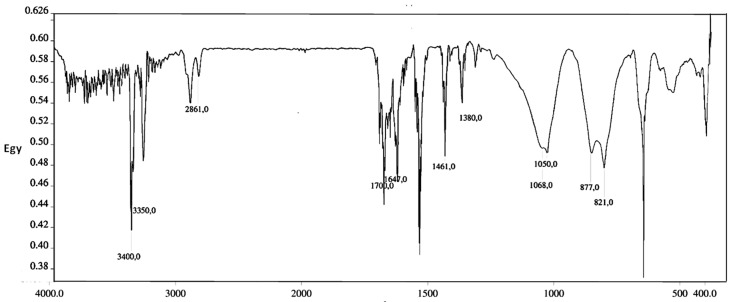
FT IR spectra mixing spectroscopic grade KBr of PEI-MWCNTs (**4**).

Evidences for the CNTs functionalization were provided by FT-IR. Epoxide ring-opening and post-functionalization of grafted nanotubes PEI-MWCNTs (**4**) were spectroscopically confirmed by NH_2_ band appeared at 3400 and 3350 cm^−1^, due to the NH_2_ symmetric stretch. Further evidence was given by signals at 1461 and 1380 cm^−1^, due to the bending of CH_2_ groups, while bands at 1068 and 1050 cm^−1^ were attributed to C–N and C–O bending (Figure 2).

**Figure 3:**
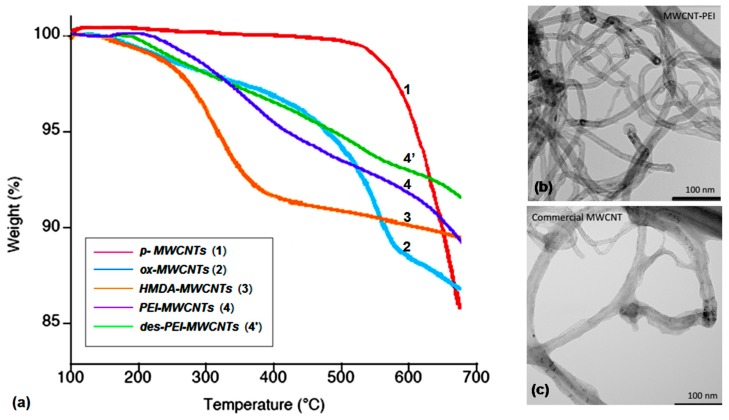
(**a**) TGA profiles of MWCNTs **1-4’**; TEM images of (**b**) PEI-MWCNTs (**4**) and (**c**) p-MWCNTs (**1**).

TGA analyses (Figure 3a) of amine-grafted nanotubes **3** and **4** revealed similar weight loss percentages of 14.4% and 14.0%, respectively, thus indicating a quite similar amine loading on the nanotubes surface. As control experiments, both p-MWCNTs **1** and grafted PEI-MWCNTs **4**’ obtained after an adsorption/desorption cycle (namely des-PEI-MWCNTs) were subjected to the same analysis.

The weight loss of 12.3% for desorbed PEI-MWCNTs (**4’**) accounted for a substantial stability of grafted amines-CNTs under the CO_2_ capture conditions. Moreover, comparison of TEM images of PEI-MWCNTs **4** (Figure 3b) and p-MWCNTs **1** (Figure 3c) revealed no substantial change in morphology upon surface functionalization of p-MWCNTs.

Loading of CO_2_-philic amine groups of modified MWCNTs **3**-**4** was evaluated by means of total N-content (%), whereas their sorbent capacity was estimated in a series of adsorption/desorption experiments and compared with that of pristine p-MWCNTs (**1**) (Table 2). As expected, grafting of amine moieties onto the surface of MWCNTs increased their sorbent capacity by conjugating physical adsorption of nanotubes and chemical absorption of CO_2_-philic functionalities. Additionally, results in Table 2 confirmed that the CO_2_ capture process proved to be perfectly reversible for amine-grafted-MWCNTs **3**-**4**, for which CO_2_ could be quantitatively released at 80 °C.

On these bases, also taking into account the recent literature [47] highligting polyethylenimine as one of the greatest candidates for CO_2_ sequestration processes, PEI-grafted MWCNTs **4** showing the best capture performance was selected as co-catalysts of titania in the CO_2_ photoreduction.

### 3.2. Preparation and Characterization of Hybrid Nanocomposites Photocatalysts

**Scheme 1:**
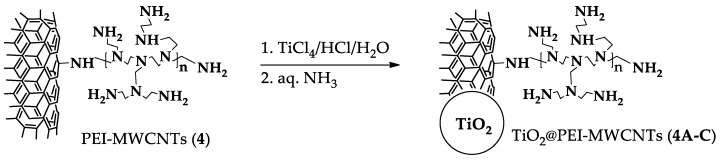
Procedure for hybrid nanocomposites preparation.

Hybrid nanocomposites photocatalysts were prepared according to a sol-gel method [14]. At this end, functionalized nanotubes **4** were suspended in a aqueous HCl solution of TiCl_4_ from which TiO_2_ was precipitated by addition of ammonia, thus forming random agglomerates by inclusion of CNTs (Scheme 1) [13]. The ensuing TiO_2_@PEI-MWCNTs composites were collected by filtration, washed with distilled H_2_O and dried at 80 °C for 24 h. For the photocatalytic experiments, a number of hybrid nanocomposites were selected (**1A**, **1C**, **4A**-**C**) using different amounts of pristine nanotubes **1** and PEI-modified- MWCNTs **4**, as listed in Table 3.

Hybridized materials were characterized by BET, XRD, XPS, FE-SEM, and UV techniques (see Supplementary Materials for details). BET analyses displayed an expected trend in which the presence of carbon nanotubes remarkably increased the surface area of composites with respect to titania (Table 3).

**Figure 4:**
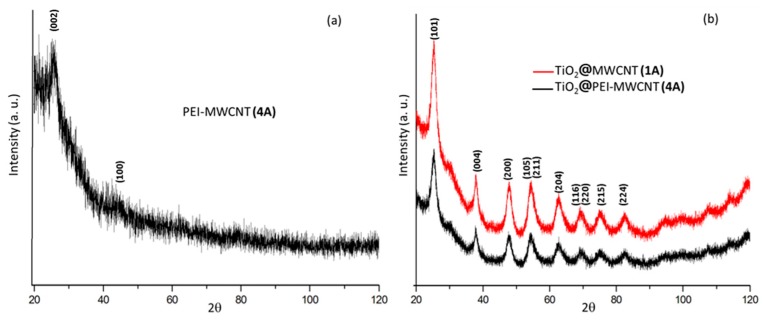

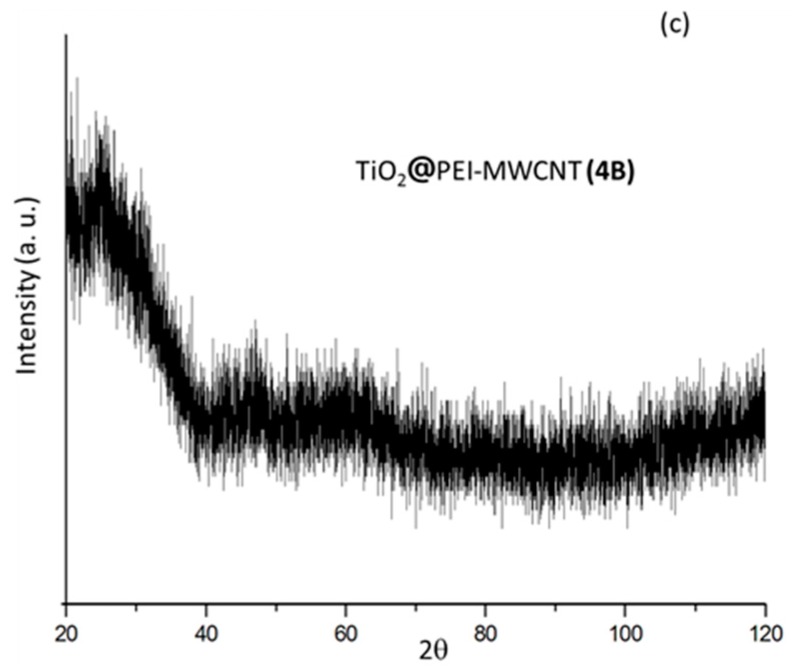
XRD patterns of samples (**a**) **4**; (**b**) **1A**; (**c**) **4A**-**B**.

XRD spectra were reported in Figure 4. The diffraction pattern of PEI-grafted MWCNTs **4** showed a very low degree of crystallinity (Figure 4a). The diffraction patterns of the two hybrid composites **1A** and **4A**, consisting of TiO_2_ with minimal amounts (2%) of carbon nanotubes, exhibited similar features of anatase phase (Figure 4b). As expected, on increasing the PEI-MWCNTs content up to 10% (composite **4B**), the anatase polymorphic form did not persist anymore (Figure 4c), most likely because the increase of the almost amorphous nanotubes hinders the agglomeration of TiO_2_ particles [14,15,16,17,18] (see Supplementary Materials for details).

XPS analyses showed the expected surface atomic concentration of composites (Table 4). Notably, from both atomic percentages and high-resolution N1s signals (Figure S3) emerged that PEI-MWCNTs-TiO_2_ composite did not undergo to a sensible variations of its surface composition after catalytic reaction. The N1s signal could be curve fitted with two components: a dominant component at 400.5 ± 0.2 eV ascribed to amino groups and a minor component centred at 402.2 ± 0.2 eV that could be due to protonated amino groups or to amino groups involved in hydrogen bonding. This is a clear evidence of photocatalyst stability under the reaction conditions.

SEM images of samples **1A** and **4C** (Figure S4) showed heterogeneous samples consisting of MWCNTs randomly dispersed into TiO_2_ agglomerates. A high level of magnification (Figure S4(D)) highlights the close interfacial contact between nanotubes and TiO_2_ which is crucial for narrowing of titania bandgap and consequently for the enhancement of catalytic activity [48].

**Figure 5:**
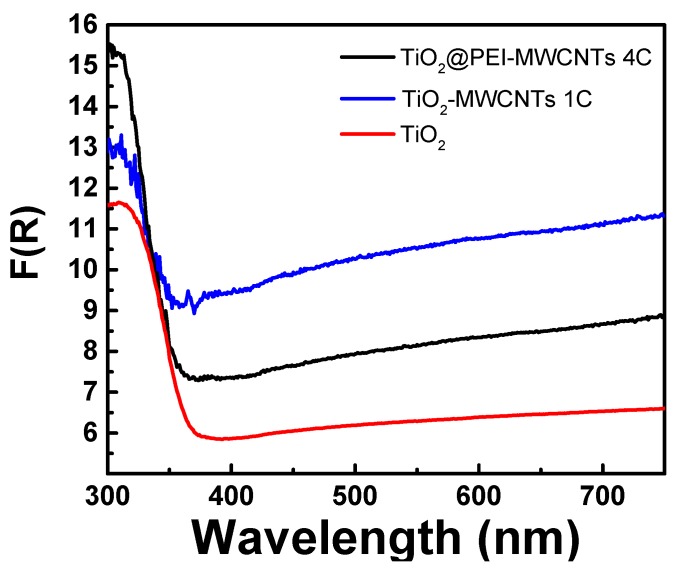
Absorbance spectra in Kubelka-Munk units of photocatalytic materials.

UV-Vis reflectance spectra of TiO_2_, TiO_2_@MWCNTs (**1C**) and TiO_2_@PEI-MWCNTs (**4C**) recorded in absorption mode are presented in Figure 5. As expeted, all hybrid materials showed a higher light absorption capability in the UV region below 400 nm, due to the characteristic absorption of TiO_2_. Remarkably, spectra of **1C** and **4C** appear to be quite similar, displaying the same red shift effect caused by the incorporation of both nanotubes alone and PEI-modified-CNTs into TiO_2_ [49,50,51]. This means that the formation of covalent bonds between MWCNTs and PEI in **4C** does not introduce significant modifications of graphite-like structure of nanotubes. However, the composite **4C** showed a more intense absorption underneath 350 nm, maybe due to the auxochrome effect (mainly n→σ* electronic transitions) of amino and hydroxyl groups linked on nanotubes surface. This effect could explain the enhanced photoreducing power displayed by composite **4C** in the photoreductionof the organic dye UBA in the UV range (see mechanistic insights paragraph).

### 3.3. Phocatalytic Experiments

Photocatalytic conditions were chosen according to the goal of this study: the development of a metal free catalyst system that mimics the natural photosynthetic process, i.e., ambient temperature, sunlight radiation and water as the hydrogen source.

As a further aim of this work, we attempted to investigate the influence of radiation source on photocatalysis using two different emitting lamps: (i) the SANOLUX lamp, displaying a higher radiance in the near UV (315–400 nm) and the Xe-Halogen lamp, possessing the major emission power in the visible range (400–700 nm, see Figure S6 in Supplemetary Materials).

Photoreduction was performed in a water phase suspension (20 mL) of the solid catalyst (with the loading in the range of 7.5–30 mg_solid.cat._/mL), and monitoring reaction parameters and species produced in both the liquid solution and in the gas phase of the reactor head space [43] (see Figure S5).

Argon was initially bubbled for 30 min, to avoid the presence of air, then CO_2_ for approximately 60 min, to saturate the reaction mixture, upon which suspension was irradiated for 5 h under vigorous stirring at room temperature. Initial pH value of solution after bubbling was found to be equal to 4.78, and raised to 5.80 after reaction.

**Scheme 2:**
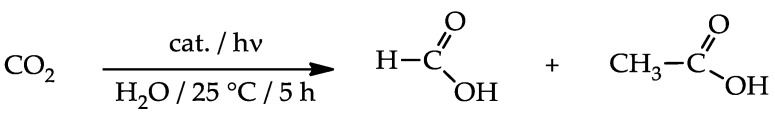
Photoreduction of carbon dioxide.

Under the chosen conditions, nanocomposites proved to be active in converting CO_2_ into formic and acetic acids (Scheme 2), while neither simple PEI-grafted nanotubes nor bare TiO_2_ displayed appreciable activity (Table 5, runs 1–2).

**Figure 6:**
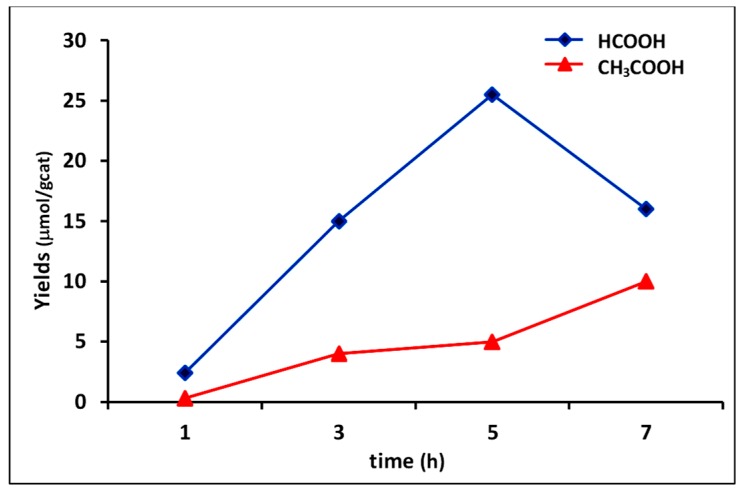
Monitoring of yields versus time in photocatalysis with composite **4C** (experimental conditions of run 7, Table 5).

The optimal irradiaton time of 5 h was ascertained by monitoring photoreduction in the first 7 h of reaction (Figure 6). Results showed that prolonged reaction times(>5 h) led to the decrease of yield in formic acid with the corresponding increment of acetic acid. A plausible explanation could be related to the partial transformation of HCOOH into CH_3_COOH by reaction with methyl radicals (CH_3_), according to the general mechanism proposed by S. Sharifnia et al. [52].

Composites **1A** and **1C** beharing unmodified MWCNTs gave formic acid as the major product (Table 5, runs 3–4). In addition, photoreducing power, especially the one leading to formic acid, increased with increasing the percentage of PEI-grafted nanotubes in the composite reaching the maximum level at about 30% (sample **4C**, Table 5, runs 5–7).

The head space gas phase was monitored during reaction by means of GC analyses using a specific Barrier Ionization Discharge (BID) detector for revealing the reaction by-products.

Sampling of the gaseous phase above the reaction solution at different time intervals showed a constant composition consisting essentially of O_2_ (2%), Argon (5%) and CO_2_ (93%). Neither CO, nor H_2_ or other gaseous by-products (methane, ethane etc.) were detected during reaction. Notably, the light source proved to have a profound influence on the reaction selectivity: Xe-Halogen lamp (emitting primarily into the visible region) privileged the formation of formic acid, while SANOLUX lamp (with a main radiance in the near UV) favoured the formation of acetic acid (Table 5, runs 8–9).

Catalyst loading affected reaction in a predictable manner. Its increment increased the light scattering, thus limiting photoreducing process. As a consequence, despite the use of a threefold amount of solid catalyst, yields remained almost unchanged (Table 5, runs 11–14).

### 3.4. Catalyst Recycling

**Figure 7:**
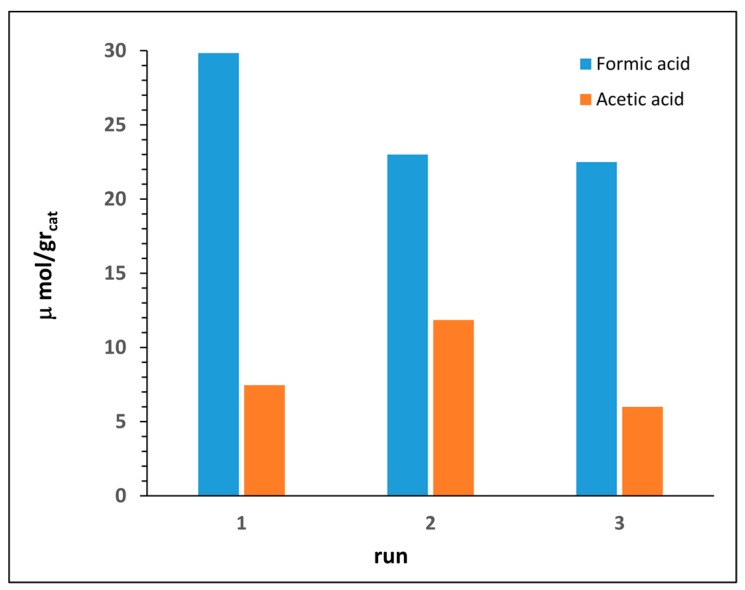
Recycling of photocatalyst nanocomposite **4C**.

Stability and robustnes of our nanocatalysts were evaluated by means of recycling experiments (Figure 7). After each run the catalyst powder was recovered by filtration, washed with distilled methanol and water, and then dried under vacuum. Results evidenced a fair to good catalyst stability, with only a little loss of efficiency observed after the first cycle.

### 3.5. Mechanistic Insights

The photocatalytic process of CO_2_ reduction follows a relative unknown and complex mechanism leading to different products at the same time. Among them, CO, CH_3_OH, HCOOH, CH_4_, HCHO, CH_3_COOH and other light hydrocarbons are mainly observed, with a selectivity affected by a great number of factors [52].

**Figure 8:**
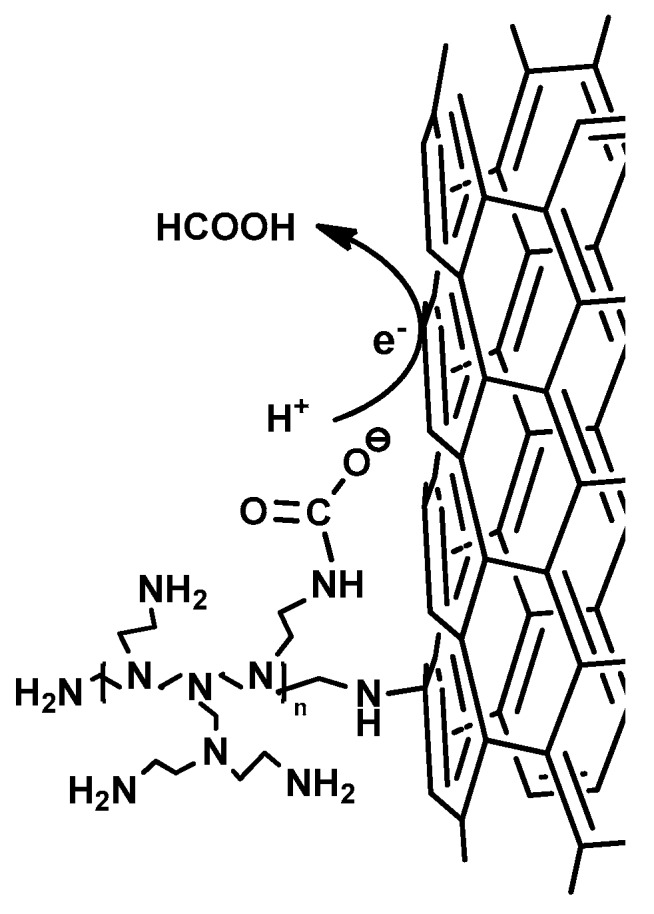
Activation of CO_2_ in photoreduction by PEI through carbamate formation.

**Figure 9:**
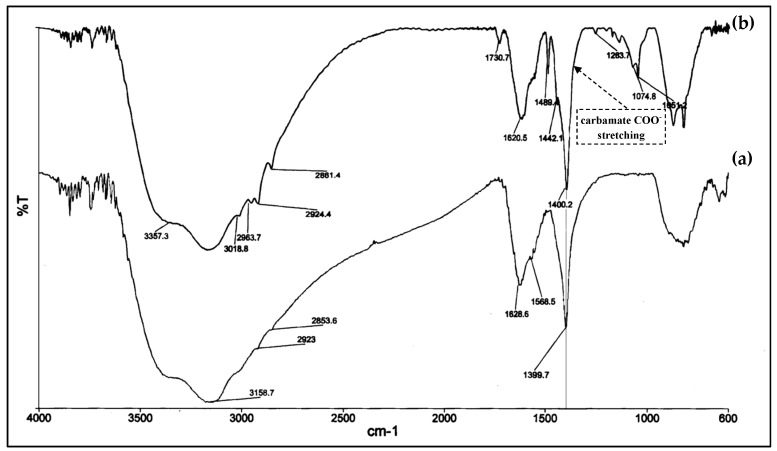
FT-IR images of TiO_2_@PEI-MWCNTs (**4C**) under photocatalytic conditions (a) before and (b) after CO_2_ bubbling.

**Figure 10:**
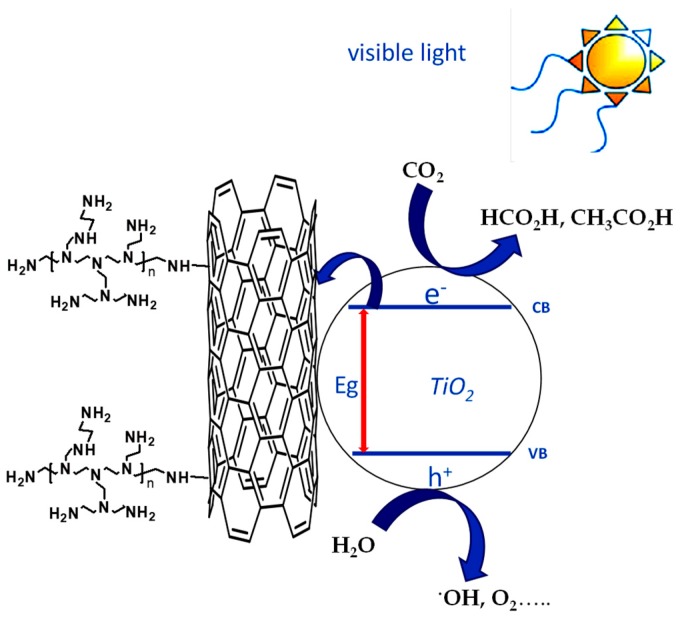
Effect of PEI conjugation on separation of photogenerated electron–hole pairs for TiO_2_@PEI-MWCNTs nanocomposite.

We do not have elements to propose specific mechanisms, but can rationalize in the following points our experimental results attempting also to explain the remarkable enhancement of activity that polyamine modification of TiO_2_/MWCNTs nanocomposites can lead in CO_2_ photoreduction:

(i) Firstly, the detection of O_2_ into the head space of photoreactor can be considered a clear evidence that water is the sacrificial reductant for CO_2_.

(ii) Secondly, the high chemisorption capacity of TiO_2_@PEI-MWCNTs (**4C**) (18 mL_CO_2__/g) is most probably the key reason for the its enhanced photoreducing ability, due to kinetic effects. Indeed, reaction of CO_2_ with NH_2_ groups forms carbamate, thus leading to the activation of carbon dioxide, because carbamate is believed to have a higher reactivity than the linear CO_2_ molecule (Figure 8) [24]. This assumption is corroborated by FT-IR analyses (Figure 9) of (**4C**) under photocatalytic conditions before and after CO_2_ bubbling, in which the peak at 1489 cm^−1^ appeared that can be attributed to the carbamate COO^−^ stretching [53,54].

(iii) Thirdly, an electronic effect of PEI could also be invoked in the UV range. It has been extensively approved that MWCNTs can narrow the band gap of TiO_2_ extending light absorption to longer wavelength region. The red shift observed in Figure 5 confirms such a hypothesis. In addition, due their graphite-like structure, MWCNTs are considered as a channel for electron storage, functioning as electron acceptors from higher conduction band of TiO_2_, thus inhibiting the recombination rate of electron-hole pairs through the transfer of electrons [24]. This is extremely crucial as the slower recombination rate leaves more holes in TiO_2_, promoting higher oxidation rate of water and thus enhancing reduction of CO_2_ (Figure 10).

In this context, it can be assumed that PEI-grafting, from one hand, does not modificate the graphite-like structure of CNTs thus allowing them to continue their action of narrowing the band gap of titania, while, form the other hand, can enhance the photocatalytic activity of TiO_2_ into the UV range due to the auxochrome effect.

**Scheme 3:**
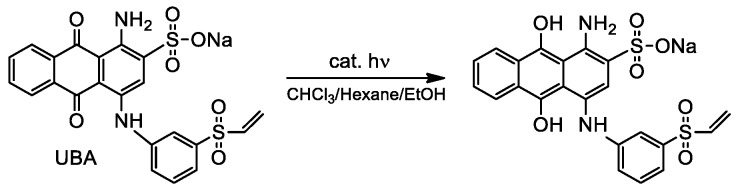
photoreduction of UBA.

Notably, lowering of crystallinity experienced by TiO_2_ as a consequence of hybridization (Figure 4c) does not influence catalytic performance, as this detrimental effect is counterbalanced by the enhancement of both the electron scavenging and CO_2_ capture capacity of nanocomposite by virtue of PEI-CNTs conjugation.

(iv) Searching for further confirmations on the effect on photocatalytic activity of PEI-conjugation with TiO_2_/MWCNTs composites, we investigated the photoreductionof the organic dye UBA, (Scheme 3) a model substrate commonly used to investigate electron transfer reactions [18,44,45]. Irradiation was performed with UV light using a mercury lamp (λ > 250 nm) and photoreduction was checked monitoring the colour change from blue (the colour of UBA anthraquinonic structure) to yellow (due to UBA reduced form).

**Figure 11:**
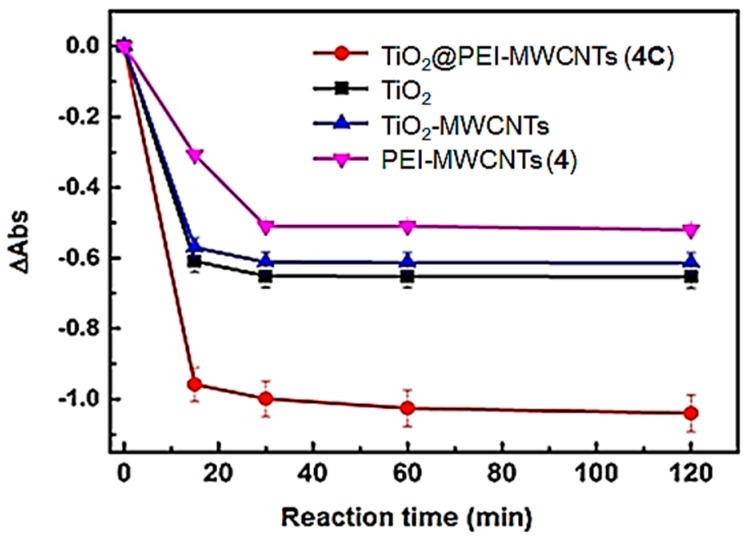
Time course of UBA bleaching monitored at 587 nm. Data are reported as mean value of three replicates ± standard deviation.

Differences in absorbance (ΔAbs) acquired at 587 nm as a function of irradiation time were reported (Figure 11) [44,45]. Results clearly evidenced a higher reducing capability of the TiO_2_@PEI-MWCNTs (**4C**) compared to the bare TiO_2_. Indeed, with **4C** as catalyst, an almost complete photoreduction of UBA was observed in the first 30 min of reaction, with the ΔAbs value of 0.98. After that, the ΔAbs value reached a plateau.

Conversely, with bare TiO_2_, the ΔAbs was detected to be 0.6 indicating that titania alone can promote UBA photoreduction up to the 60% in the first 30 min of irradiation. Next, despite prolonged irradiation the ΔAbs remained constant.

Two further control experiments were performed using PEI-MWCNTs (**4**) and TiO_2_@MWCNTs (**1C**) sample as catalysts, respectively. The former resulted in a 30% photoreduction of UBA during the first 30 min of irradiation, while the latter gave rise to 56% of UBA photoreduction, displaying a reduction capacity comparable with that of bare TiO_2_ and lower than that observed for TiO_2_@PEI-MWCNTs (**4C**). This beneficial role played by the functionalization with PEI could be explained with the auxochrome effect in the UV region exerted by amino and hydroxyl groups of PEI-modified composites (clearly visible in the UV spectrum of Figure 5), and is in accordance with the accepted principles “the higher the light absorption, the more the photoexcited electron–hole pairs” or also “the higher the reflectance the higher the activity” [55].

In addition, the disappointing results of UBA reduction assisted by PEI-MWCNTs (**4**), pointed out that an efficient UBA photoreductionis promoted only in the presence of TiO_2_, especially upon its interaction with MWCNTs efficiently mediated by PEI. Remarkably, the sample TiO_2_-MWCNTs (**1C**) showed a lower photocatalytic efficiency respect to the TiO_2_@PEI-MWCNTs (**4C**), despite they have a comparable specific surface area (Table 3: 299.2 m^2^/g and 304.2 m^2^/g, respectively). Such a behaviour further highlights the beneficial role played by the functionalization of MWCNTs with the PEI in the achievement of a highly photoactive nanocomposite.

(v) Finally, a special attention deserves the photoreduction selectivity towards the two main photoreduction products HCOOH/CH_3_COOH. Results in Table 5 clearly show that formation of HCOOH increases with increasing of PEI-grafted MWCNTs percentage in the nanocomposite, with the best yields reached with 30% ca. of carbon dopants (catalyst **4C**, Table 5, runs 7–8, 14). This trend is in line with the above mentioned assumptions.

More difficult is the rationale for the formation of CH_3_COOH. This product is more rarely observed, even if is the major product in water suspension [56], like in our case, or using TiO_2_ doped with metals like Cu, Rh and Ru [56,57,58]. From results in Table 5 emerged that acetic acid formation depends on the nature of light source, with highest value reached using SANOLUX UV lamp (Table 5, run 9). This result indicates that formation of acetic acid is favoured by the UV-component of radiation and seems to be in accordance with the auxochrome effect of PEI into the UV region (Figure 5).

We do not have other elements explaining this phenomenon, and further efforts are needed to unravel the complex mechanistic aspects underlying the photocatalytic activity of these new materials.

## 4. Conclusions

In summary, we have found that polyethylenimine (PEI) anchored onto multiwalled carbon nanotubes can strongly enhance photocatalytic performances of TiO_2_/MWCNTs composites, rendering these materials efficient photocatalysts for CO_2_ conversion into formic and acetic acids. The simultaneous presence of PEI and CNTs is essential for catalysis, as demonstrated by the high sorbent capacity of TiO_2_@PEI-MWCNTs (4C) (18 mL/g) that enables both CO_2_ capture and activation, most probably via carbamate formation. Bleaching experiments corroborate in part the beneficial role of PEI in enhancing the photoreducing power of TiO_2_/MWCNTs composites in the UV range by means of a plausible auxochrome effect.

To the best of our knowledge, this is the first example in the literature in which amines-grafted CNTs materials can function as TiO_2_ co-catalysts for converting CO_2_ into carboxylic acids. We hope this work opens new avenues for carbon recycling using renewable sources.

## Figures and Tables

**Table 1:** Preparation of nanocomposites **1A**, **1C** and **4A**-**C**.

Nanocomposite	MWCNTs	Amount of MWCNTs (mg)	TiCl_4_/HCl Mother Solution (mL)	Weight of Nanocomposite (g)	% (*w*/*w*) of PEI-MWCNTs in the Hybrid ^a^
TiO_2_ (blank)	–	–	250	–	–
TiO_2_@MWCNTs (**1A**)	p-MWCNTs (**1**)	20	250	1.0056	2.0%
TiO_2_@MWCNTs (**1C**)	p-MWCNTs (**1**)	500	500	1.6651	30%
TiO_2_@PEI-MWCNTs (**4A**)	PEI-MWCNTs (**4**)	20	250	1.1152	1.8%
TiO_2_@PEI-MWCNTs (**4B**)	PEI-MWCNTs (**4**)	200	500	1.9508	10.3%
TiO_2_@PEI-MWCNTs (**4C**)	PEI-MWCNTs (**4**)	500	500	1.6197	30.9%

^a^ Evaluated based on the weights of suspended MWCNTs and recovered nanocomposites.

**Table 2:** CO_2_ adsorption/desorption ability of amine-grafted MWCNTs.

Amine-Grafted MWCNTs	N-Content (%) by elem. Analysis	Adsorbed CO_2_ at 25 °C (mL·g^−1^) ^a^	Desorbed CO_2_ at 80 °C (mL·g^−1^) ^b^
p-MWCNTs 1	–	11	11
HMDA-MWCNTs 3	1.01	24	24
PEI-MWCNTs 4	2.63	35	35

^a^ Adsorption time 6 h. ^b^ Desorption time 3 h (see Supplementary Materials for details).

**Table 3:** Hybrid nanocomposites preparation.

Hybrid ^a^	% (w/w) of PEI-MWCNTs in the Hybrid	BET Surface Area (m^2^/g)
1A (control)	2.0% ^b^	176.7
1C (control)	30% ^b^	299.2
4A	1.8%	161.8
4B	10.3%	327.3
4C	30.9%	304.2

^a^ See Table 1 for details. ^b^ Unmodified MWCNTs were hybridized as control composites.

**Table 4:** XPS analyses of hybrid nanocomposites.

Surface Atomic Concentrations (%)				
Sample	C	O	N	Ti
PEI-MWCNTs (**4**)	85.8 ± 0.5	6.8 ± 0.2	7.4 ± 0.2	–
TiO_2_@PEI-MWCNTs (**4C**)	55.2 ± 0.6	30.6 ± 0.6	3.7 ± 0.3	10.5 ± 0.3
TiO_2_@PEI-MWCNTs (**4C**) *	59.8 ± 0.6	27.6 ± 0.5	3.3 ± 0.2	9.3 ± 0.3

* After reaction.

**Table 5:** Catalytic performance of TiO_2_@PEI-MWCNT hybrids in the photoreduction of CO_2_.

Run	Cat.	% (*w*/*w*) of PEI-MWCNTs in Hybrid	Cat. Loading (mg/mL) ^a^	Light Source ^b^	Photocatalytic Products (μmol·g^−1^ Cat.) ^c^		
					HCOOH	CH_3_CO_2_H	Total Carbonaceous
1	PEI-MWCNTs (**4**)	–	7.5	H/S	0.21	0.01	0.22
2	TiO_2_	–	7.5	H/S	1.73	0.01	1.74
3	**1A**	2.0% ^d^	7.5	H/S	2.67	0.38	3.05
4	**1C**	30.0% ^d^	7.5	H/S	5.80	2.73	8.53
5	**4A**	1.8%	7.5	H/S	4.05	4.30	8.35
6	**4B**	10.3%	7.5	H/S	13.01	8.49	21.50
7	**4C**	30.9%	7.5	H/S	29.84	7.46	37.30
8	**4C**	30.9%	7.5	H	25.50	4.98	30.48
9	**4C**	30.9%	7.5	S	3.99	23.00	26.99
10	**4C ^e^**	30.9%	7.5	H/S	0.086	0.012	0.096
11	**1A**	2.0%	25	H/S	0.76	0.15	0.91
12	**4A**	1.8%	25	H/S	0.51	0.48	0.99
13	**4B**	10.3%	25	H/S	1.11	2.86	3.97
14	**4C**	30.9%	25	H/S	16.50	2.44	18.94

^a^ Catalyst loading is 150 mg or 500 mg dispersed in 20 mL of water solution. ^b^ S = SANOLUX HRC UV-VIS lamp 300 W; H = RADIUM Xe-Halogen lamp 400 W. Irradiation time 5 h. ^c^ Estimated by ionic chromatography. HCOOH and CH_3_COOH formation was neither detected in the dark conditions nor without photocatalyst. ^d^ Unmodified MWCNTs were hybridized as control composites. ^e^ Without CO_2_.

## Supplementary Materials

The following are available online at http://www.mdpi.com/1996-1944/11/2/307/s1. Detailed XRD, XPS, SEM analyses, CO_2_ photoreduction apparatus, Spectra of emission lamps (Figures S1–S6). **Figure S1**: Schematic representation of apparatus for measuring CO_2_ adsorption/desorption ability, **Figure S2**: XRD patterns of samples **4**, **1A**, **4A-B**, **Figure S3: High-resolution N1s signal**, **Figure S4**: SEM image of (A) sample TiO_2_@MWCNTs **1A**. SEM images of sample TiO_2_@PEI-MWCNTs **4C** at (B) 2 μm, (C) 200 nm and (D) 100 nm of magnification level, **Figure S5**: Schematic of CO_2_ photoreduction apparatus, **Figure S6**: Emission spectra of (a) HRC UV-VIS lamp 300 W (Sanolux) and (b) Xe-Halogen lamp 400 W (Radium).
