# Supplementary material for: TiO2@PEI-Grafted-MWCNTs Hybrids Nanocomposites Catalysts for CO2 Photoreduction
Source: Materials (Basel). 2018 Feb 20;11(2):307. doi: 10.3390/ma11020307 (PMC5849004; doi:10.3390/ma11020307)
Supplement: Supplementary file 1 [file materials-11-00307-s001.pdf]

Supplementary materials

TiO_2@PEI-grafted-MWCNTs Hybrids Nanocomposites Catalysts for CO_2 Photoreduction

Caterina Fusco,[1,*] Michele Casiello,[2] Lucia Catucci,[2] Roberto Comparelli,[3] Pietro Cotugno,[4] Aurelia Falcicchio,[5] Francesco Fracassi,[2] Valerio Margiotta,[3] Anna Moliterni,[5] Francesca Petronella, [3] Lucia D'Accolti,[1,2,*] and Angelo Nacci,[1,2,*]

Contents:

Measuring the CO_2 adsorption/desorption ability of amine-grafted MWCNTs (Fig. S1) .... page 2

Detailed XRD analyses of hybrids nanocomposites (Fig. S2) .... page 3

Detailed XPS analyses with High-resolution N1s signal (Figure S3) .... page 4

SEM analyses of **1A** and **4C** nanocomposites (Fig. S4) .... page 5

Schematic of CO_2 photoreduction apparatus (Fig. S5) .... page 6

Emission spectra of Lamps (Fig, S6).. page 7

Measuring the CO_2 adsorption/desorption ability of amine-grafted MWCNTs

A gas burette was used to evaluate the adsorption/desorption ability of amine-grafted MWCNTs. The gas volume of CO_2 was measured in a thermostated schlenk tube connected with the burette (Fig. S1). The sample was degassed under vacuum for 2 hours at room temperature (25°C). When adsorption step began, a CO_2 flow was maintained for 6 hours at 25°C. When adsorption step was complete, a flow of inert N_2 was introduced into the flask to remove residual carbon dioxide. Then, the temperature was increased to 80 °C for 3 hours to accomplish the desorption step.

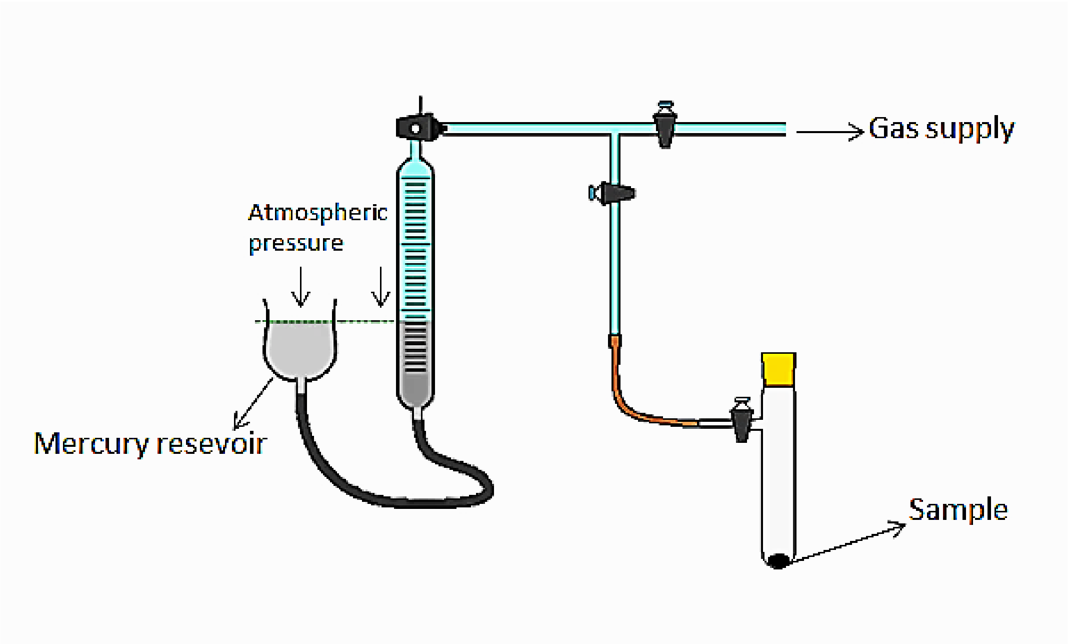

Figure S1. Schematic representation of apparatus for measuring CO_2 adsorption/desorption ability

Detailed XRD analyses of hybrids nanocomposites

The diffraction pattern of PEI-grafted MWCNTs **4** displayed two probable low intensive and widened peaks (fig. S4a), indicating a very low degree of crystallinity of the sample. Due to its similarity with the diffraction pattern of purified MWCNTs described in the paper by Xia *et al*. [14], the two broad peaks can be associated to the (0 0 2) and (1 0 0) reflections of the MWCNTs. The diffraction patterns of the two samples **1A** and **4A**, consisting of TiO_2 with minimal amounts (2%) of carbon nanotubes, showed similar features and were processed by the qualitative analysis software *QUALX2.0* that identified the anatase TiO_2 phase.

In fig. 4b the Miller indices of anatase TiO_2 reflections associated to the major diffraction peaks are shown. As expected, on increasing the PEI-MWCNTs content up to 10% (sample **4B)**, the anatase polymorphic form did not persist anymore (fig. 4c), most likely because the increase of the almost amorphous nanotubes hinders the agglomeration of TiO_2 particles.

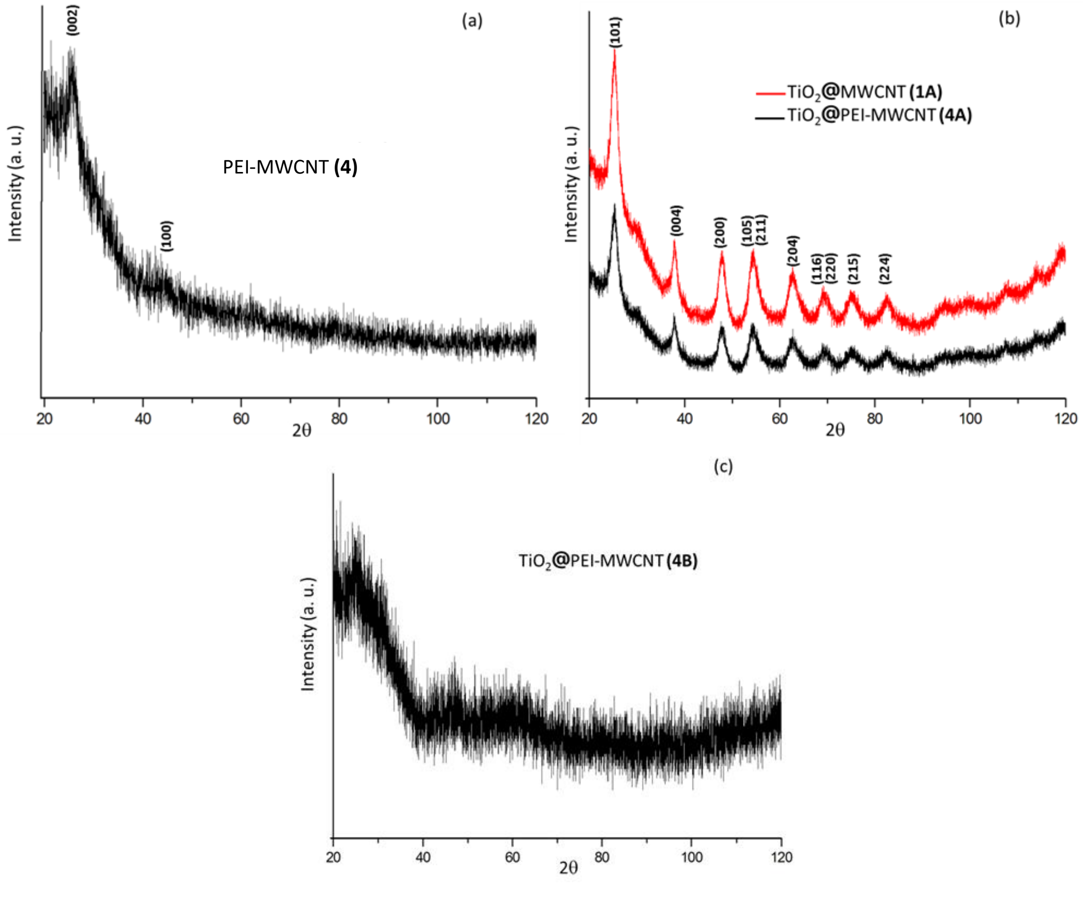

Figure S2. XRD patterns of samples **4**, **1A**, **4A-B**.

Detailed XPS analyses with High-resolution N1s signal

The N1s signal could be curve fitted with two components a dominant component at 400.5 ± 0.2 eV ascribed to amino groups and a minor component centred at 402.2 ± 0.2 eV that could be due to protonated amino groups or to amino groups involved in hydrogen bonding (Fig. S2).

<table>
<tr><td>PEI-MWCNTs</td><td>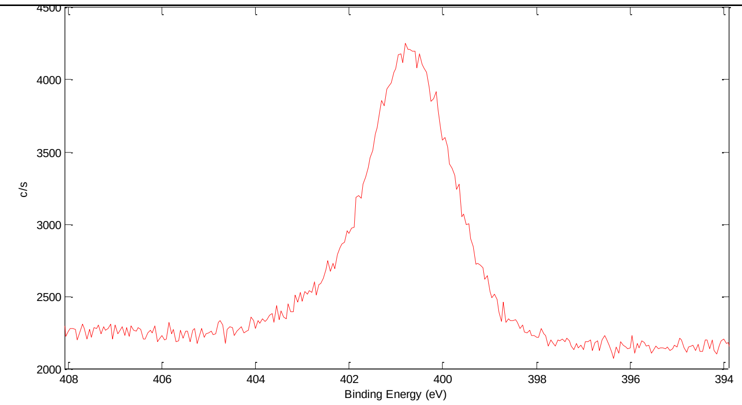
</td></tr>
<tr><td>PEI-MWCNTs-TiO_2</td><td>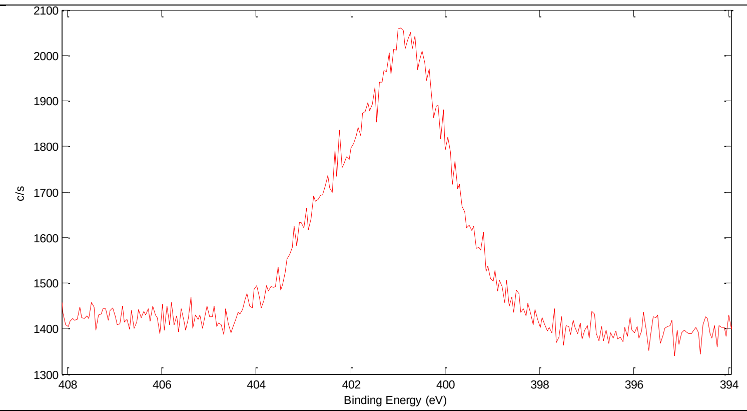
</td></tr>
<tr><td>PEI-MWCNTs-TiO_2/after reaction</td><td>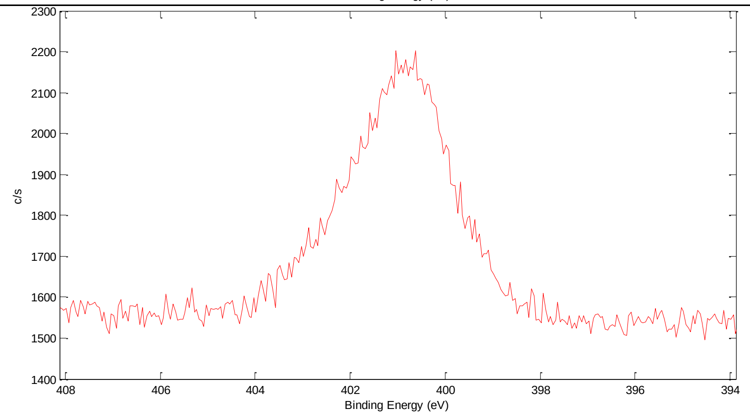
</td></tr>
</table>

Figure S3. High-resolution N1s signal

SEM analyses of 1A and 4C nanocomposites

SEM image of **1A** and **4C** (Fig. S4) shows heterogeneous samples consisting of carbon nanotubes randomly dispersed into TiO_2 agglomerates. In particular, image of **4C** at the highest level of magnification (Fig. S4D) better highlights the close interfacial contact between CNTs and TiO_2.

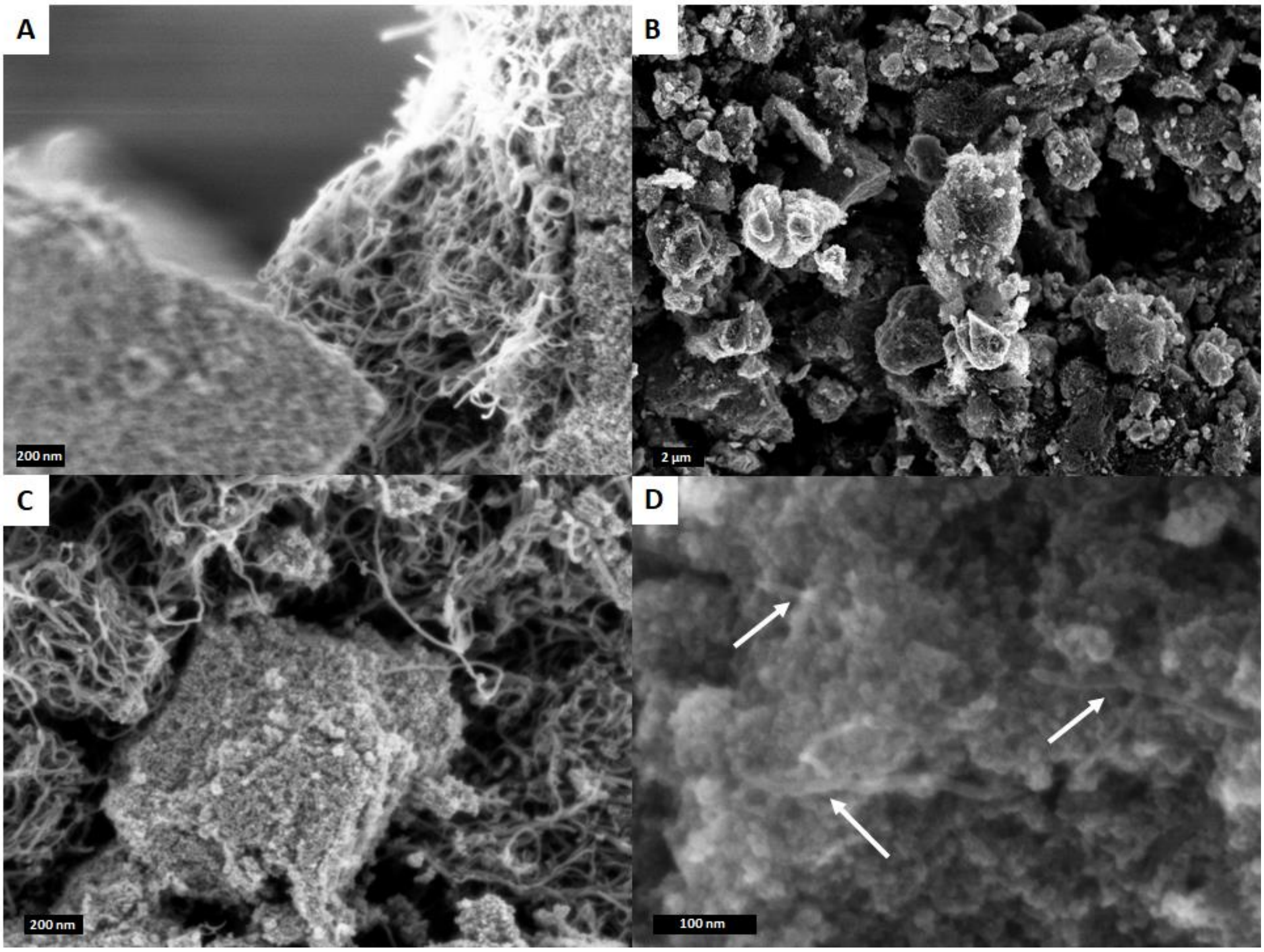

Figure S4. SEM image of (A) sample TiO_2@MWCNTs **1A**. SEM images of sample TiO_2@PEI-MWCNTs **4C** at (B) 2 µm, (C) 200 nm and (D) 100 nm of magnification level. In particular, blank arrows in the latter image better highlights the close interfacial contact between CNTs and TiO_2

Schematic of CO_2 photoreduction apparatus

Apparatus for photocatalytic experiments consisted of a three necked Pyrex batch photoreactor of cylindrical shape equipped with a jacket for cooling water circulation and ports in its upper section useful for gas inlet (and outlet), sampling and pH and temperature measurements. Two lamps were placed in proximity of the reactor (5-6 cm): HRC UV-VIS lamp 300W (Sanolux) and Xe-Halogen lamp 400W (Radium)

In a typical procedure, in 20 ml of an aqueous suspension of photocatalyst, argon was bubbled for 30 minute, to avoid the presence of air, and then CO_2 for approximately 60 minutes before switching on the lamps. Two catalyst amounts chosen for optimization experiments were 150 and 500 mg corresponding to loadings of 7.5 g/ml and 25 g/ml, respectively. The initial measured pH value was 4.58 and temperature inside the reactor was kept at approximately 25 °C by a continuous water circulation through the photoreactor jacket. The photoreactivity runs lasted 5.0 h and products were detected by Ion Cromatography.

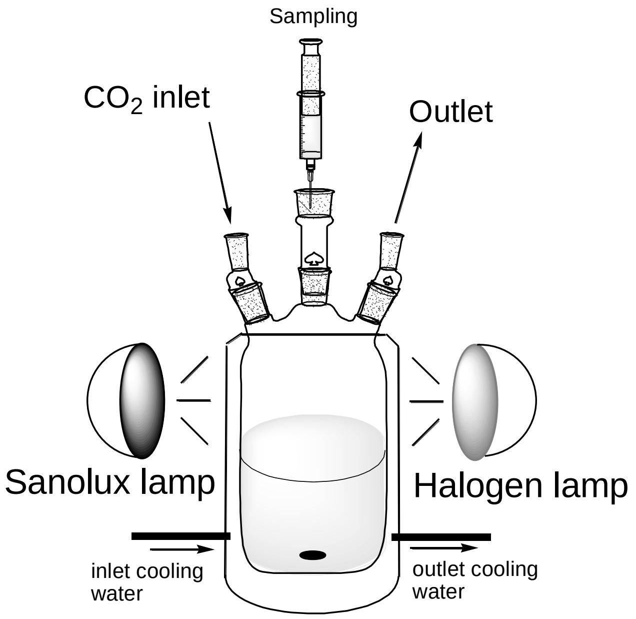

Figure S5. Schematic of CO_2 photoreduction apparatus.

Emission spectra of Lamps

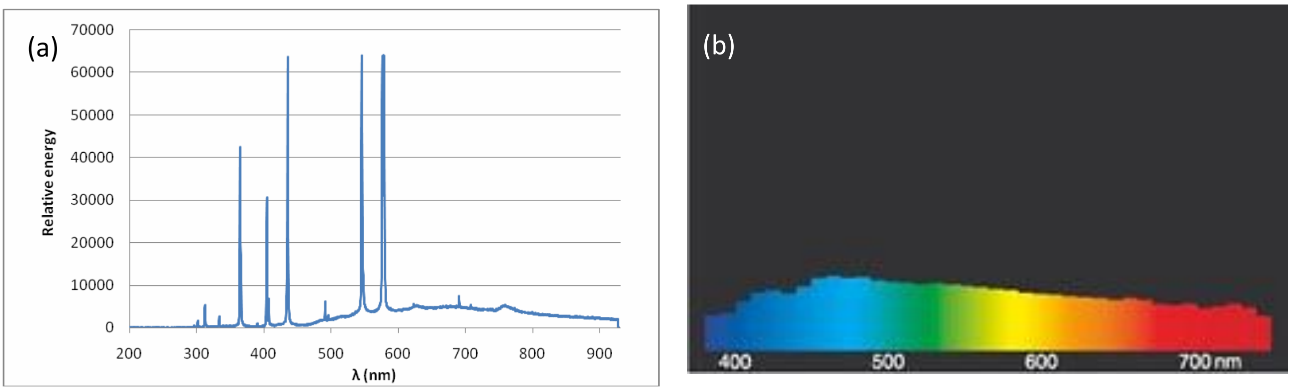

Figure S6. Emission spectra of (a) HRC UV-VIS lamp 300W (Sanolux) and (b) Xe-Halogen lamp 400W (Radium)

Lamps	315-400 nm<br>IrradianceW/m^2	400-700 nm<br>Irradiance on PAR (Photosyntetically Active Radiation)<br>μmol/m^{2}s
Sanolux	46.80x10^3	7.4x10^3
Xe-Halogen	7.16x10^3	14.31x10^3

Emission Intensity in photoreactor:

Lamps	315-400 nm<br>Irradiance W/m^2	400-700 nm<br>IRRADIANZA nella regione delle radiazioni per la misura del PAR (Photosyntetically Active Radiation)<br>μmol/m^{2}s
Sanolux/Xe	14.98x10^3	5.32x10^3
Sanolux	2.20x10^3	0.968x10^3
Xe-Halogen	1.41x10^3	4.19x10^3
